# Targeting the NAD Salvage Synthesis Pathway as a Novel Therapeutic Strategy for Osteosarcomas with Low NAPRT Expression

**DOI:** 10.3390/ijms22126273

**Published:** 2021-06-10

**Authors:** Natasja Franceschini, Jan Oosting, Maud Tamsma, Bertine Niessen, Inge Briaire-de Bruijn, Brendy van den Akker, Alwine B. Kruisselbrink, Ieva Palubeckaitė, Judith V. M. G. Bovée, Anne-Marie Cleton-Jansen

**Affiliations:** Department of Pathology, Leiden University Medical Center, 2333 ZA Leiden, The Netherlands; n.franceschini@lumc.nl (N.F.); J.Oosting@lumc.nl (J.O.); mtamsma@gmail.com (M.T.); bertineniessen@hotmail.com (B.N.); I.H.Briaire-de_Bruijn@lumc.nl (I.B.-d.B.); B.E.W.M.van_den_Akker@lumc.nl (B.v.d.A.); A.B.Kruisselbrink@lumc.nl (A.B.K.); I.Palubeckaite@lumc.nl (I.P.); J.V.M.G.Bovee@lumc.nl (J.V.M.G.B.)

**Keywords:** NAMPT, NAPRT1, differentiation, cancer metabolism, osteosarcoma, NAD synthesis

## Abstract

For osteosarcoma (OS), the most common primary malignant bone tumor, overall survival has hardly improved over the last four decades. Especially for metastatic OS, novel therapeutic targets are urgently needed. A hallmark of cancer is aberrant metabolism, which justifies targeting metabolic pathways as a promising therapeutic strategy. One of these metabolic pathways, the NAD+ synthesis pathway, can be considered as a potential target for OS treatment. Nicotinamide phosphoribosyltransferase (NAMPT) is the rate-limiting enzyme in the classical salvage pathway for NAD+ synthesis, and NAMPT is overexpressed in OS. In this study, five OS cell lines were treated with the NAMPT inhibitor FK866, which was shown to decrease nuclei count in a 2D in vitro model without inducing caspase-driven apoptosis. The reduction in cell viability by FK866 was confirmed in a 3D model of OS cell lines (*n* = 3). Interestingly, only OS cells with low nicotinic acid phosphoribosyltransferase domain containing 1 (NAPRT1) RNA expression were sensitive to NAMPT inhibition. Using a publicly available (Therapeutically Applicable Research to Generate Effective Treatments (TARGET)) and a previously published dataset, it was shown that in OS cell lines and primary tumors, low NAPRT1 RNA expression correlated with NAPRT1 methylation around the transcription start site. These results suggest that targeting NAMPT in osteosarcoma could be considered as a novel therapeutic strategy, where low NAPRT expression can serve as a biomarker for the selection of eligible patients.

## 1. Introduction

**Figure 1:**
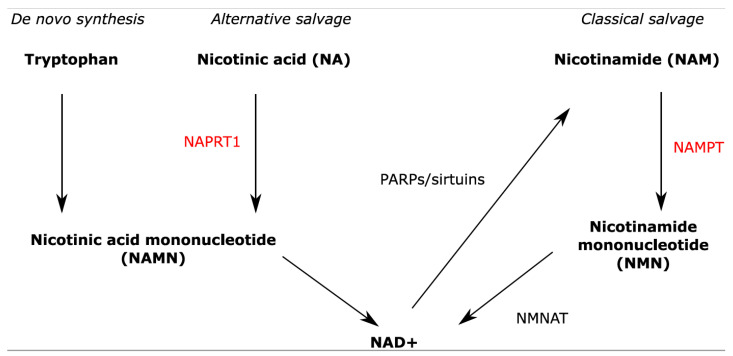
Simplified overview of the NAD+ synthesis pathway. NAD+ is synthesized by de novo synthesis, the alternative salvage pathway, or the classical salvage pathway. NAMPT and NAPRT1 are the rate-limiting enzymes of the classical salvage and alternative salvage pathways, respectively. NMNAT = Nicotinamide mononucleotide adenylyltransferase 1.

Osteosarcoma is the most common high-grade malignant tumor of the bone, primarily diagnosed in children and adolescents [1]. Histologically, conventional osteosarcoma is characterized by the presence of osteoblast-like neoplastic cells that produce osteoid [1]. The current treatment strategy consists of a combination of (neo)adjuvant chemotherapy and surgery, which has greatly improved the outcome for osteosarcoma patients since its introduction, with a 5-year overall survival rate of 71% [2]. However, in the last four decades, the survival rates have not improved [2] despite the high number of in vitro studies using cell lines [3]. Targeting the aberrant metabolism which hallmarks many tumors [4,5] is a likely option for osteosarcoma. A previously published study illustrated the importance of metabolism in OS as metastatic OS cells were found to be highly metabolically active, and therefore targeting metabolism in OS can be a potential novel therapy [6]. Furthermore, it was shown that both the inhibition of mTOR—a key regulator of metabolism, and the inhibition of 3-phosphoglycerate dehydrogenase (PHGDH)—the rate-limiting enzyme of serine biosynthesis, attenuated cell proliferation in OS cells and can therefore serve as a novel therapeutic target [7,8,9]. The NAD+ synthesis pathway is a metabolic pathway often upregulated in tumors as cancer cells need to maintain a high level of NAD+ required for cell survival processes [10,11]. NAD+ is an essential co-enzyme central to many metabolic processes such as aerobic glycolysis, the citric acid cycle, oxidative phosphorylation, fatty acid metabolism, and anti-oxidant metabolism [12]. NAD+ can be synthesized de novo, or via the alternative salvage or classical salvage pathway [12,13] (Figure 1). In the alternative salvage pathway, NAD+ synthesis is catalyzed by the enzyme nicotinic acid phosphoribosyltransferase domain containing 1 (NAPRT1), whereas in the classical salvage pathway, it is catalyzed by the enzyme nicotinamide phosphoribosyltransferase (NAMPT). Cancer cells rely mostly on the classical NAD+ synthesis pathway as de novo synthesis from amino acids is inefficient and the alternative pathway is often inactive due to the lack of expression of NAPRT1 [13,14]. In osteosarcoma, NAMPT is overexpressed as compared to that found in normal bone [15]. Targeting NAMPT could therefore potentially be a novel therapeutic option for osteosarcoma patients; consequently, the main aim of this study is to test the sensitivity of osteosarcoma cells to NAMPT inhibition.

In a recent study, a connection between NAMPT and osteogenic differentiation was discovered [16], in which NAMPT expression increased during osteogenic differentiation in osteoblasts. As the hallmark of osteosarcoma is the production of the osteogenic matrix (osteoid) by tumor cells [1], we further investigated whether NAMPT inhibition would be affecting osteogenic differentiation and mineralization in osteosarcoma.

In this study, it was discovered that cell viability of osteosarcoma cells decreased after treatment with the NAMPT inhibitor FK866, in both 2D cultured and 3D cultured cells. More importantly, only OS cells with a low expression of NAPRT1, and as such depending more on the classical salvage pathway for NAD+ synthesis, were sensitive to NAMPT inhibition. Low NAPRT1 expression in primary tumor samples of osteosarcoma correlated with the methylation of the NAPRT1 promotor. Therefore, low NAPRT expression could serve as a potential biomarker for the selection of osteosarcoma patients who could benefit from treatments that inhibit NAMPT.

## 2. Results

### 2.1. Osteosarcoma Cells Show Variable Sensitivity to NAMPT Inhibition

**Figure 2:**
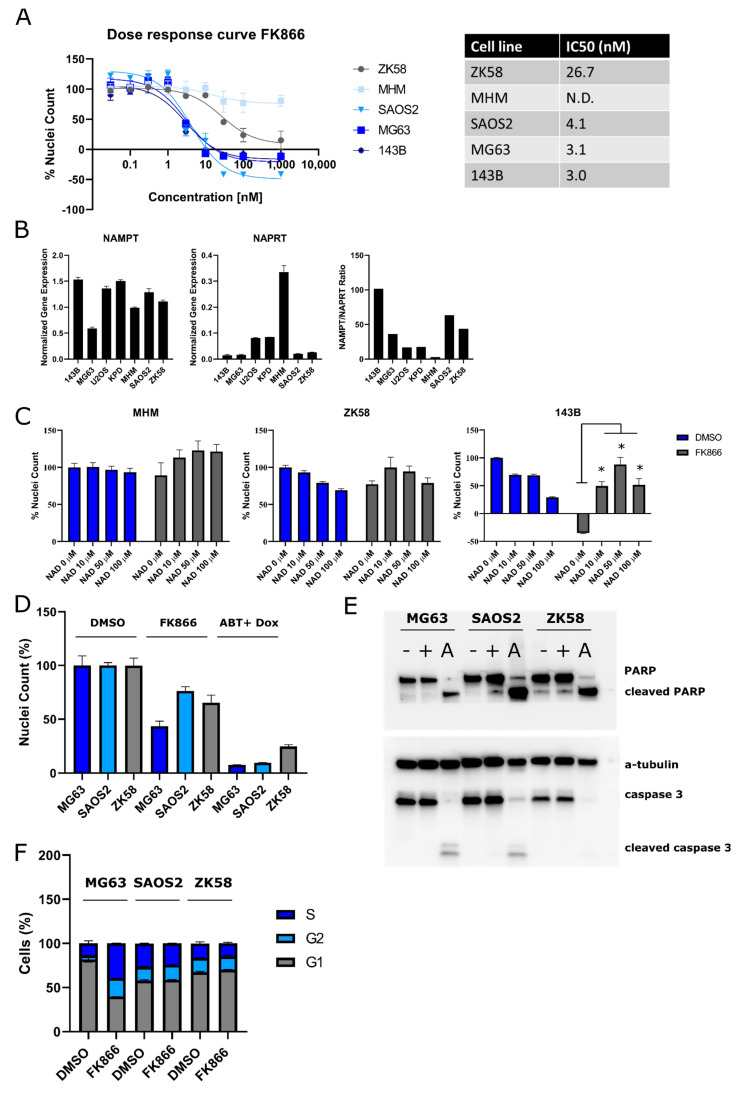
**The** NAMPT inhibitor FK866 decreases cell viability in 2D cultured osteosarcoma cell lines. (**A**) Osteosarcoma cell lines were treated for 72 h with the NAMPT inhibitor FK866, after which relative cell viability and IC_50_ values were determined. IC_50_ could not be determined for MHM (N.D.). Data points represent the mean of two experiments performed in triplicate ± standard deviation. (**B**) Gene expression of NAMPT and NAPRT1 in osteosarcoma cell lines. (**C**) NAD+ rescues the inhibitory effect of the NAMPT inhibitor FK866 (10 nM) on cell viability. Bars represent the mean of two experiments performed in triplicate ± standard deviation. * = *p* < 0.05 (**D**) OS cells treated for 48 h with FK866 (5 nM (SAOS2 and MG63) or 60 nM (ZK58)) reduced cell viability. Cells treated with ABT-737 (5 µM) and doxorubicin (1 µM) were used as a positive control for the induction of apoptosis [17]. Bars represent the mean of two experiments performed in triplicate ± standard deviation. (**E**) Western blot for cleaved PARP and cleaved caspase 3 after 48 h treatment with FK866 (+; 5 nM (SAOS2 and MG63) or 60 nM (ZK58)), or 0.1% DMSO (−). No increased apoptosis was observed in OS cell lines. OS cells treated with ABT-737 (5 µM) and doxorubicin (1 µM) (A) were used as a positive control for the induction of apoptosis [17]. Experiments were performed two times and one representative blot is shown. (**F**) Cell cycle analysis after 48 h treatment with FK866 (5 nM (SAOS2 and MG63) or 60 nM (ZK58)) showed that treatment induced changes in cell cycle progression in MG63 cells, but not in SAOS2 and ZK58. Bars represent the mean of two experiments ± standard deviation.

To assess whether NAMPT inhibition affects nuclei count and cell viability in osteosarcoma, five OS cell lines—MG63, MHM, SAOS, 143B, and ZK58—were treated for 72 h with the NAMPT inhibitor FK866 (Figure 2A). There was no marked difference between nuclei count and cell viability (Figure S1A). All OS cell lines showed a dose-dependent decrease in nuclei count after NAMPT inhibitor treatment, with IC_50_ values ranging from 3.0 to 26.7 nM, except for MHM which showed very limited sensitivity to NAMPT inhibition. There was marked variability in sensitivity to NAMPT inhibition: in order to define a biomarker for the NAMPT inhibitor sensitivity of OS cell lines, RNA expression levels of NAMPT and NAPRT1 as well as the NAMPT/NAPRT1 ratio were analyzed in a panel of seven OS cell lines. NAMPT and NAPRT1 expressions were highly variable in OS cell lines, as were NAMPT/NAPRT1 expression ratios (Figure 2B). Interestingly, MHM, the OS cell line which showed very limited sensitivity to NAMPT inhibition, showed the lowest NAMPT/NAPRT1 ratio and the highest NAPRT1 expression as compared to cell lines ZK58, SAOS2, 143B, and MG63.

To assess whether the effect of NAMPT inhibition on nuclei count was specifically due to the targeting of the NAD+ classical salvage pathway, treatment with the NAMPT inhibitor FK866 was supplemented with NAD+ in three OS cell lines with high (143B), medium (ZK58), or low (MHM) sensitivity to FK866. Although NAD+ itself reduced nuclei counts, NAD+ treatment rescued the inhibitory effect of the NAMPT inhibitor FK866 partially in OS cell lines 143B and ZK58 (Figure 2C). MHM cells showed limited sensitivity to FK866 treatment and an addition of NAD+ increased nuclei count in cells treated with FK866, although this was not statistically significant. To investigate whether FK866 reduced nuclei count by inducing apoptosis—as this was previously suggested to be how FK866 induced cell death [18]—OS cells that were sensitive to FK866 (MG63, SAOS2, ZK58) were treated with FK866. Apoptosis was subsequently measured by western blot of cleaved caspase 3 and cleaved PARP. OS cells treated for 48 h with FK866 showed decreased nuclei count (Figure 2D), but the same cells did not show any cleaved caspase 3 or cleaved PARP on western blot, indicating that FK866 did not induce apoptosis in these cells (Figure 2E). To investigate if FK866 has an effect on cell cycle progression, OS cells were treated with FK866. Marked changes in cell cycle progression were observed after FK866 treatment in the OS cell line MG63 as the percentage of cells in the G2-phase decreased, while the percentage of cells in the S-phase and the G1-phase increased (Figure 2F). However, two other OS cell lines, SAOS2 (FK866 sensitive) and ZK58 (relatively sensitive), did not show any changes in cell cycle progression after the FK866 treatment.

### 2.2. 3D Cultured Osteosarcoma Cells Are Also Sensitive to NAMPT Inhibition

**Figure 3:**
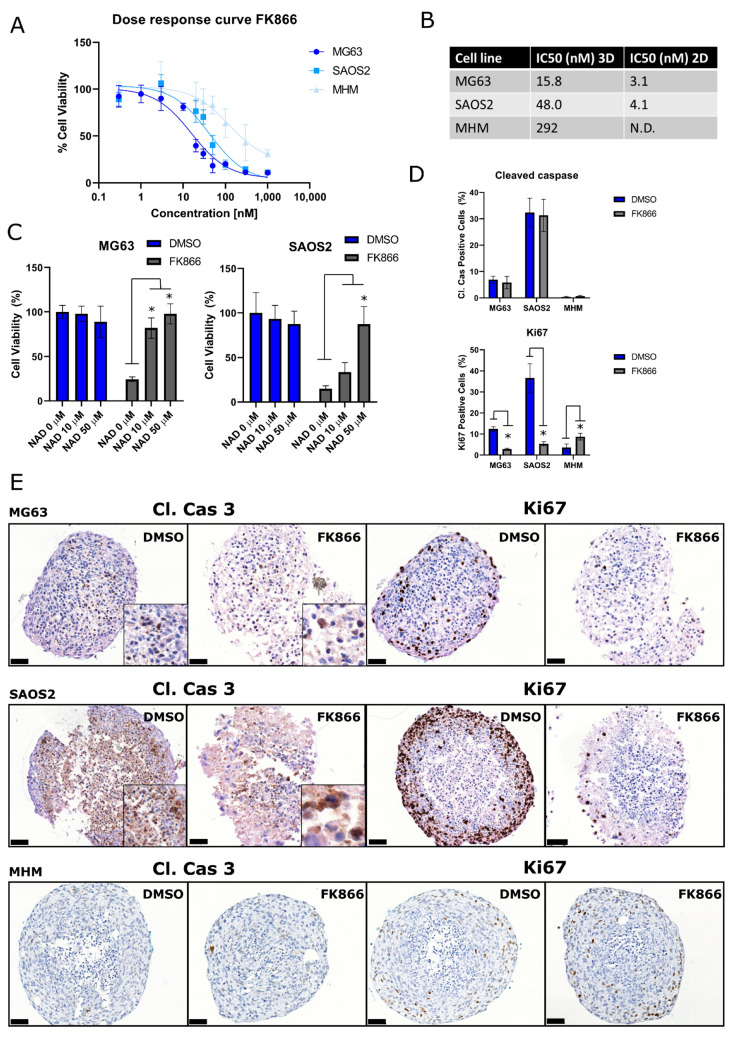
The NAMPT inhibitor FK866 decreases cell viability in 3D cultured osteosarcoma cell lines. (**A**) Osteosarcoma multi-cellular tumor spheroids were treated for 72 h with the NAMPT inhibitor FK866, after which relative cell viability and (**B**) IC_50_ values were determined. IC_50_ value for MHM could not be determined (N.D.). Data points represent the mean of two experiments performed ± standard deviation. (**C**) NAD+ rescues the inhibitory effect of the NAMPT inhibitor FK866 (30 nM) on cell viability in MG63 and SAOS2. Bars represent the mean of one experiment performed in triplicate ± standard deviation. * = *p* < 0.05 (**D**) Quantification and (**E**) representative images of the cleaved caspase 3 and Ki67 stained sections of 3D cultured osteosarcoma cells treated with DMSO or FK866 (30 nM). Scale bar represents 50 µm. * = *p* < 0.05.

As 3D cultured cells are often more representative of the in vivo situation compared to 2D cultured cells, multi-cellular tumor spheroids (MCTS) of the OS cell lines MHM, MG63, SAOS2 were generated and treated for 72 h with the NAMPT inhibitor FK866 to determine cell viability. 3D cultured OS cell lines were sensitive to the NAMPT inhibitor treatment (Figure 3A). The MHM cell line, which did not show sensitivity to the NAMPT inhibitor in 2D, also showed limited sensitivity in our 3D model compared to the other OS cell lines, with an IC_50_ of 292 nM (Figure 3B). In our 3D model of OS cell lines MG63 and SAOS2, NAD+ treatment also rescued the effect of FK866 treatment on cell viability (Figure 3C). Immunohistochemical staining of the multi-cellular tumor spheroids for Ki67 and Cleaved Caspase 3 showed no changes in apoptosis (Figure 3D,E), which was in line with the results obtained from the caspase 3 and PARP western blot in 2D (Figure 2E). However, a reduction of proliferating cells was observed in SAOS2 and MG63 after FK866 treatment (Figure 3D,E), whereas the insensitive MHM showed a slight increase of Ki67 positive cells.

### 2.3. FK866 Showed a Variable and Time-Dependent Effect on Osteogenic Differentiation in the OS Cell Line ZK58

**Figure 4:**
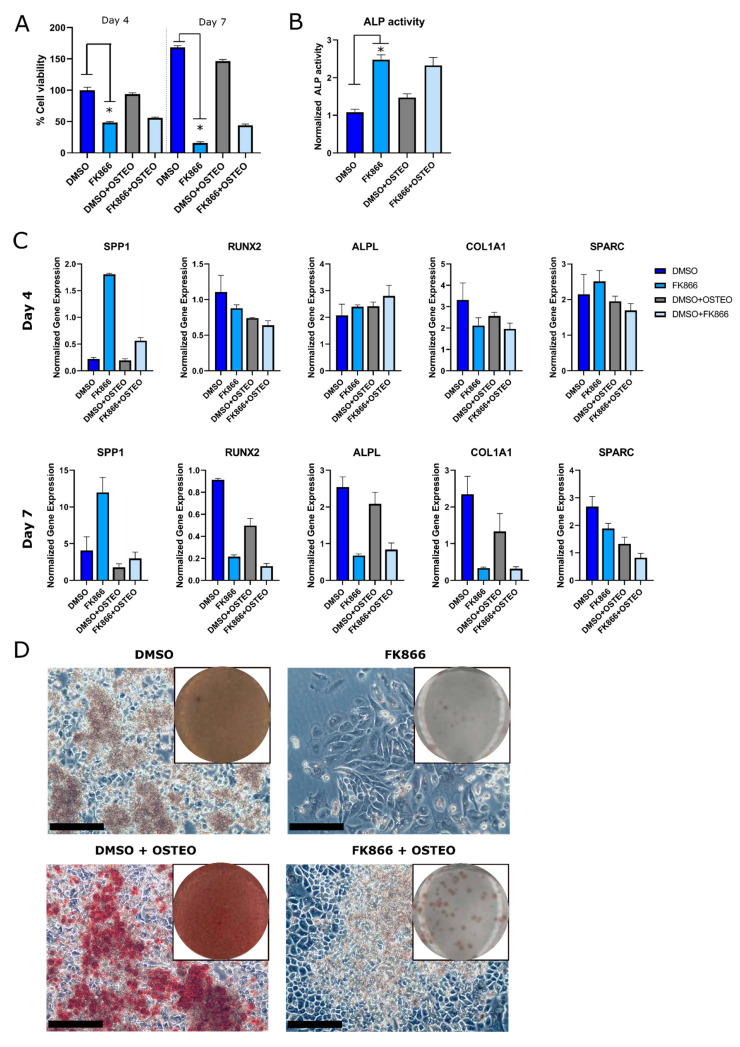
Effect of FK866 on osteogenic differentiation in OS cell line ZK58 is dependent on the duration of treatment. All bars represent the mean of two experiments in triplicate ± standard deviation. * = *p* < 0.05. (**A**) Only the FK866 treatment (25 nM), and not the osteogenic medium, affects cell viability. (**B**) ALP enzymatic activity increases after four days of FK866 treatment (25 nM). (**C**) Osteogenic gene expression marker *SPP1* is upregulated after four or seven days of FK866 treatment (25 nM), whereas markers *ALPL*, *COL1A1*, *SPARC,* and *RUNX2* are downregulated after seven days of treatment. (**D**) Mineralization was inhibited in FK866 (25 nM) treated OS cells, after 21 days of osteogenic differentiation. One representative image is shown for each condition. Scale bar represents 100 µm.

A positive link between osteogenic differentiation in osteoblasts and NAMPT expression has recently been suggested [16]. Therefore, to determine whether osteogenic differentiation is affected by NAMPT inhibition in OS, we used the OS cell line ZK58, the only cell line in our panel with a known osteogenic differentiation potential [19]. ZK58 was treated for four or seven days with FK866 in an osteogenic differentiation medium. Only FK866 and not the osteogenic medium reduced cell viability in ZK58 (Figure 4A). Osteogenic differentiation was determined after four or seven days of treatment with FK866, by measuring ALP activity and the gene expression levels of a panel of osteogenic markers (COL1A1, ALPL, RUNX2, SPP1, SPARC). FK866 increased ALP activity, irrespective of the presence or absence of the osteogenic differentiation medium after four days of treatment (Figure 4B). The gene expression of several osteogenic markers was not affected by FK866 treatment after the four days; however, the in the case of SPP1, gene expression was increased by FK866 treatment irrespective of the culture medium (Figure 4C). After seven days of treatment with FK866, changes in gene expression occurred. The osteogenic marker SPP1 was still upregulated after FK866 treatment, while other markers (COL1A1, ALPL, RUNX2) were downregulated (Figure 4C). The downregulation of osteogenic markers (SPP1, RUNX2, SPARC) was also observed in MCTS of ZK58 treated for seven days with FK866 (Figure S1B). To investigate the effect of NAMPT inhibition on mineralization, OS cells were treated for 21 days with FK866, after which mineralization was assessed with an Alizarin Red staining. FK866-treated cells showed no visible Alizarin Red staining around the cell population that remained, indicating that no mineralization had occurred (Figure 4D).

### 2.4. NAPRT Expression in Osteosarcomas Correlates Inversely with Methylation of the NAPRT Promotor

**Figure 5:**
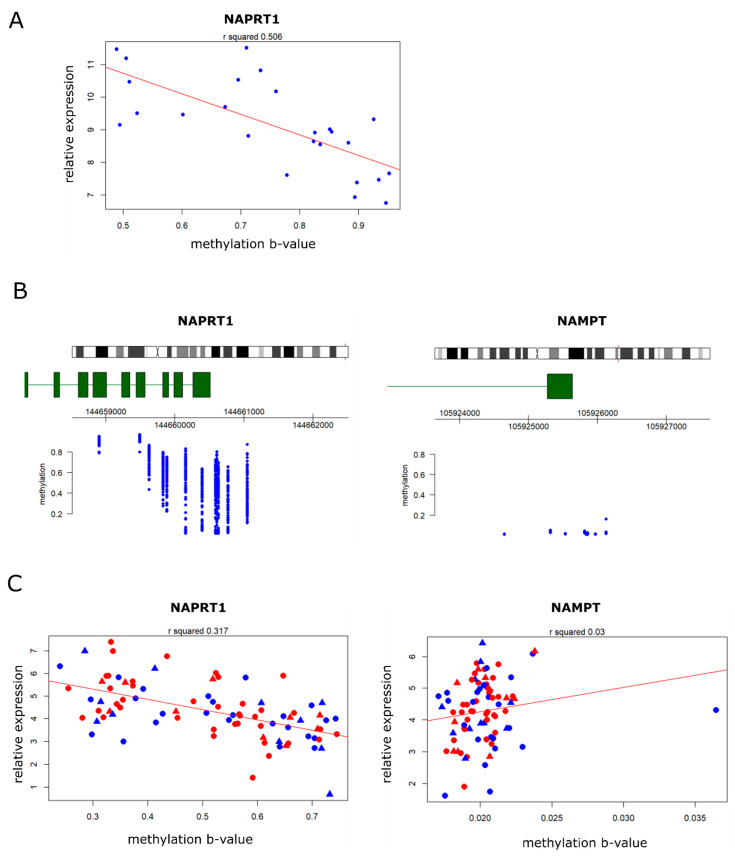
NAPRT promotor methylation correlates with low NAPRT1 expression in osteosarcoma. (**A**) In OS cell lines, methylation of NAPRT1 correlates with the low expression of NAPRT1. (**B**) Transcription start site of NAPRT1, and not NAMPT, has areas of methylation in a subset of osteosarcoma tumors. (**C**) High methylation correlates with the low expression of NAPRT1 in osteosarcomas. NAMPT gene expression does not correlate with the methylation status. Red = male, blue = female, circle = non-metastatic, triangle = metastatic.

OS cell lines with low NAPRT1 expression exhibited the highest sensitivity to NAMPT inhibition. In addition, the low expression of NAPRT1 was previously found to be correlated with NAPRT1 promotor hypermethylation in chondrosarcomas [20]. Thus, the methylation levels of NAPRT in OS cell lines were compared with the gene expression of NAPRT1. In a previous study, methylation arrays have been performed in osteosarcoma cell lines [21]. Using this dataset, NAPRT1 promotor methylation was shown to correlate with decreased NAPRT1 expression in OS cell lines (Figure 5A).

NAPRT1/NAMPT expression and methylation were further investigated in a publicly available dataset of the Therapeutically Applicable Research to Generate Effective Treatments (TARGET) (https://ocg.cancer.gov/programs/target (accessed on 22 February 2021).) initiative, comprising 86 osteosarcomas and includes data from whole genome RNA expression deep sequencing analyses and DNA methylation arrays. Methylation levels around the NAPRT1 transcription start site were determined; osteosarcomas showed variable levels of methylation around the transcription start site (Figure 5B). These results demonstrate that NAPRT1 and NAMPT expression in primary tumors is variable. There was no correlation between NAMPT and NAPRT1 expression levels in primary tumors (Figure S1C). The methylation level of NAPRT1 correlated inversely with NAPRT1 expression levels (r^2^ = 0.317), but there was no correlation with parameters such as sex and the presence of metastasis (Figure 5C). In contrast, the NAMPT promotor was not methylated in any of the samples, and therefore no correlation between methylation and expression levels could be observed (r^2^ = 0.03) (Figure 5B,C).

## 3. Conclusions and Discussion

Despite the large number of next-generation sequencing studies identifying targets in osteosarcoma [22,23,24] and the high volume of in vitro studies [3], no novel therapeutic options for osteosarcoma have been brought to the clinic since the introduction of conventional chemotherapy. In this study, we investigated whether targeting the NAD+ metabolic pathway in osteosarcoma could be a novel therapeutic strategy for osteosarcoma patients. It was shown that osteosarcoma cell lines, both in 2D and 3D cell culture models, are sensitive to NAMPT inhibition by FK866.

We investigated whether NAMPT inhibition affected nuclear count and cell viability in osteosarcoma cell lines by inhibiting apoptosis, or by attenuating cell proliferation. In the current study, it was shown that apoptosis was not induced in OS cells after treatment with FK866. To investigate cell proliferation, cell cycle analysis and Ki67 staining was performed on FK866-treated OS cells. Ki67 staining showed that the number of proliferating cells was reduced by FK866 in two out of three OS cell lines, suggesting that FK866 inhibited cell proliferation. Cell cycle analysis showed that the percentage of MG63 cells in the G2 phase decreased after FK866 treatment, which could suggest a G2 arrest. The observed increased percentage of cells in the S-phase suggests an arrest in the S-phase in the middle of DNA duplication, given the decrease of nuclei count observed after FK866 treatment. Interestingly, only MG63 showed changes in cell cycle progression; the two other FK866-sensitive OS cell lines, SAOS2 and ZK58, did not exhibit such changes. So far, it remains unclear whether the changes in the cell cycle observed in MG63 are an exception. The effect of FK866 is variable among OS cell lines, which is in line with previous studies in literature that have investigated the mechanism of how FK866 reduces cell viability. Previous studies have shown that FK866 does not have an effect on cell cycle distribution [18], or on the contrary, that FK866 inhibits cell cycle progression [25]. Similarly, it was demonstrated that FK866 either induced apoptosis ([18]) or that it did not induce caspase-driven apoptosis in cancer cell types with rapid ATP turnover [25,26]. Instead, these cells undergo oncosis-mediated cell death, characterized by cell swelling upon cell death [26,27]. Other mechanisms, such as autophagy, have also been suggested [28]. Taken together, the current study underlines the fact that there is currently no consensus in the field on the exact mechanism of FK866 and that future studies should be performed to investigate this in more detail.

Osteosarcoma is characterized by tumor cells that produce an osteogenic matrix. As earlier studies suggested that NAMPT inhibits osteogenic differentiation in murine osteoblast and bone marrow-derived mesenchymal stem cells [16,29], we investigated whether NAMPT also affected osteogenic differentiation in osteosarcoma cells. We indeed found that FK866 treatment inhibited mineralization in osteosarcoma cells, but this was not reflected in the RNA expression patterns and may also be attributed to the loss of cell viability. In contrast, the early osteogenic marker ALP was increased after a short-term FK866 treatment and may indicate that inhibition of differentiation is mainly induced after a longer treatment with FK866.

We showed here for the first time that osteosarcoma cell lines are sensitive to NAD+ depletion by using NAMPT inhibition. In the sarcoma field, previous studies suggest that NAMPT inhibition could also be a potential novel therapeutic strategy in chondrosarcoma and Ewing sarcoma [20,25]. In chondrosarcoma, it was reported that low NAPRT1 expression correlated with increased sensitivity [20]. This is in line with the current study, where NAMPT inhibition was only effective in osteosarcoma cell lines with low NAPRT1 expression. These cancer cells solely depend on NAMPT for NAD+ supply and therefore NAMPT inhibition will cause synthetic lethality in these cells. As the balance between the NAD synthesis pathways (de novo, classical, or alternative) is different in cancer cells as compared to normal tissue, targeting NAMPT can hence be a novel therapeutic strategy. Not only osteosarcoma but also other types of cancer generally lack NAPRT1 expression [30,31,32]. In line with our results, it was previously shown that in gastric cancer cells with low NAPRT expression, cells were the most sensitive to NAMPT inhibition [33].

In the current study NAMPT inhibition was tested in both 2D and 3D cultured OS cells. It was demonstrated that multi-cellular tumor spheroids have a lower sensitivity to FK866 as compared to 2D cultured cells. It was previously shown that tight intercellular contacts can cause MCTS to have poor drug penetration [34], which could also explain the reduced sensitivity to FK866 in MCTS in the current study. An advantage of using MCTS as a model system is that these spheroids express features that occur in solid tumors, such as regions of hypoxia and necrosis [35]. In a previously published study, an increase of apoptosis was observed in MCTS as compared to 2D cultured cells [34]. Indeed, we observed apoptotic cells mainly in the core of spheroids, with a highly proliferative outer ring. Thus, it is suggested that MCTS are more representative, also in terms of metabolism [36], compared to 2D cultured cells. This makes OS MCTS an attractive model for further pre-clinical (metabolic) drug testing.

Despite promising results from this study, early clinical trials that have used FK866 in the same concentration range as what we have used (1–10 ng/mL) [37], showed dose-limiting hematological toxicities in patients with other cancer types [32,37]. The toxic effects are not surprising, as healthy tissue expresses both NAMPT and NAPRT1. Therefore, future trials should investigate the possibility of the co-administration of a NAMPT inhibitor with nicotinic acid (NA) to maintain high NAD levels in healthy tissue, or focus on patients in which NAPRT1 expression in the tumor is low in order to limit the dose of the NAMPT inhibitor needed. Pre-clinical studies indeed showed that mice in which the NAMPT inhibitor was co-administrated with NA rescued mortality and limited toxicities. Moreover, this co-administration strategy did not affect anti-tumor activity in xenograft models [38,39,40]. Likewise, a combination therapy including the currently used chemotherapeutic agents should be considered.

The status of NAPRT1 expression could be a good biomarker to predict sensitivity to NAMPT inhibitor treatment. We identified that low NAPRT1 expression correlated with high promotor methylation. Our results are in line with a previous study, which concluded that low NAPRT1 expression by promotor methylation could act as a predictive biomarker for NAMPT inhibition [41]. The current study demonstrated that in most OS cell lines, NAPRT1 RNA expression was low, whereas NAMPT expression was high. In primary tumor samples, the OS the expression of NAPRT1 and NAMPT is variable; however, a subset of patients demonstrates low NAPRT1 in their tumors. With these results combined, it is highly likely that a subgroup of osteosarcoma patients that could benefit from NAMPT inhibition exists. However, future studies should explore co-administration with nicotinic acid or conventional chemotherapy. Furthermore, future studies should investigate if the use of lower doses of the NAMPT inhibitor in tumor tissue expressing low NAPRT1, by using in vivo models, could limit toxicities.

## 4. Material and Methods

### 4.1. Cell Culture

For 2D cell culture, the osteosarcoma cell lines KPD, MHM, MG63, ZK58, SAOS2, U2OS, and 143B were cultured in RPMI 1640 medium (Gibco, Invitrogen Life-Technologies, Scotland, UK), supplemented with 10% Fetal Bovine Serum (Gibco). Cell lines were retrieved from the EuroBoNet consortium [42]. For 3D cell culture, osteosarcoma cell lines MHM, MG63, and ZK58 were cultured in αMEM (Gibco) supplemented with 10% FBS. To create multi-cellular tumor spheroids (MCTS) (protocol adapted from [43]), cells were suspended in a medium containing methylcellulose (0.24% (*w*/*v*)) dissolved in DMEM, and were seeded in 1% agarose coated 96-well plates for seven days before the start of an experiment. All cells were cultured in a humidified incubator, with 5% CO_2_ and at 37 °C. Cell lines were regularly STR-profiled using the GenePrint 10 system kit (Promega, Madison, WI, USA) and tested for mycoplasm.

### 4.2. Drug Treatment

Cells were seeded and treated after 24 h (2D cell culture) or after seven days (3D cell culture) with 0.1% DMSO as a non-treated sample or FK866 (F8557, Sigma-Aldrich, Saint Louis, MO, USA, dissolved in DMSO) in concentrations ranging from 0.1–1000 nM. For NAD+ rescue experiments, NAD+ (10127965001, Sigma-Aldrich, dissolved in RPMI 1640) was added at the same time as FK866 (10 nM for 2D culture experiments, 30 nM for 3D culture experiments), with a concentration of 10, 50, or 100 µM. Dose range was determined based on a previous study [20]. After 72 h of treatment, nuclear count or cell viability was determined. For nuclear count, cells were fixed with 4% formaldehyde, stained with 2 µg/mL Hoechst (H1399, Invitrogen Life Technologies), and nuclei were counted with the Cellomics ArrayScan VTI HCS 700 and HCS Studio Cell Analysis Software (Thermo-Fisher Scientific, Waltham, MA, USA). For cell viability assays, MCTS or 2D cultured cells were incubated with the Presto Blue cell viability reagent (A13262, Invitrogen Life Technologies) for 90 min (MCTS) or 60 min (2D cultured cells) and measured using a microplate reader (Infinite M Plex, Tecan Group Ltd., Zürich, Switzerland). After a read-out of MCTS, pellets were fixed with 4% formaldehyde containing Alcian Blue (1:200) and paraffin embedded. Cell viability was determined relative to untreated control. Each datapoint was corrected for background reads and normalized to cell number at Day 0 (before treatment) to correct for growth rate in each cell line, as described in [44]. Dose response curves were created using the GraphPad Prism version 8 (GraphPad Software, La Jolla, CA, USA). Dose response studies were performed at least two times in triplicate.

### 4.3. Cell Cycle Analysis

MG63, SAOS2, and ZK58 cells were seeded in 6-well plates and treated for 48 h the next day with 5 (MG63 and SAOS2) or 60 nM (ZK58) of FK866, or 0.1% DMSO. Drug dose was defined as the IC75 determined in a 72 h exposure in 2D. Adherent cells were trypsinized and collected with supernatant. Cells were centrifuged, washed with PBS, and fixed in cold methanol for at least 20 min, as described in [45]. Cells were stained for 30 min with DAPI in PBS/1% Bovine serum albumin/0.05% Tween and stored at 4 °C overnight. At least 10,000 single cell events were measured with the NucleoCounter NC-250 (Chemometec, Lillerød, Denmark), and analyzed using Winlist 3D Version 8, and Modfit Version 4.1.7 (Verity Software House, Topsham, ME, USA).

### 4.4. Western Blotting

Whole cell Hot-SDS lysates were obtained as described previously [46] for MG63, SAOS2, and ZK58. Cells were treated with 0.1% DMSO, FK866 (5 nM (MG63 and SAOS2), 60 nM (ZK58)), or ABT-737 (5 µM) and doxorubicin (1 µM) for 48 h, as a positive control for the induction of apoptosis [17]. The supernatant of the treated cells was collected in the same lysate sample. Protein concentrations of lysates were determined with the Bio-rad DCTM protein assay kit (5000111, Bio-rad, Hercules, CA, USA) according to the manufacturer’s protocol and measured with a microplate reader (Infinite M Plex, Tecan Group Ltd., Zürich, Switzerland). Sample loading, blotting, and quantification were performed, as previously described [46]. Blots were stained for PARP (1:1000, clone 46D11, Cell Signaling Technology, Leiden, The Netherlands), caspase 3 (1:1000, clone 8G10, Cell Signaling Technology), and gel-loading control α-tubulin (1:30,000, clone DM1A, Sigma-Aldrich).

### 4.5. Immunohistochemistry

Sections (4µm) of paraffin-embedded multicellular tumor spheroids were made; after deparaffinization and rehydration, these were used for immunohistochemical staining for Ki67 (1:1600, clone D2H10, Cell Signaling) and cleaved caspase 3 (Cleaved caspase 3 (Asp175), 1:800, Cell Signaling). Antigen retrieval was performed by incubating sections in citrate buffer (10 mM, pH 6) for 10 min and cooling down for 2 h. Sections were incubated with primary antibody overnight at 4 °C. The next day, sections were incubated with the BrightVision one step detection system poly-HRP anti-mouse/rabbit (VWRKDPVO110HRP, Immunologic, WellMed B.V., Duiven, The Netherlands) for 30 min at room temperature. Sections were washed with PBS and DAB+ Chromogen (K3468, Dako, Agilent Technologies, Carpinteria, CA, USA) was added to each slide for 10 min. Slides were counterstained with hematoxylin, dehydrated, and mounted. For quantification of the stained sections, the percentage of Ki67 or cleaved caspase 3 positive cells was determined using QuPath Software v.0.2.3 on three different sections on each slide [47].

### 4.6. Osteogenic Differentiation

The OS cell line ZK58 was seeded at 5000 cells/cm^2^ for osteogenic differentiation. One day after seeding, αMEM medium supplemented with 10% FBS was added, containing either 0.1% DMSO or FK866 (25 nM), with or without osteogenic differentiation compounds (β-glycerophosphate (5 mM, Sigma-Aldrich), dexamethasone (0.1 µM, Sigma-Aldrich), and ascorbate-2-phosphate (0.15 mM, Sigma-Aldrich)). Four or seven days after the addition of the osteogenic differentiation medium, cell viability was determined with Presto Blue. ALP activity was determined after four days of differentiation by lysing of cells with PBS/Triton 0.1% and incubating with pNPP (P7998, Sigma-Aldrich) for 4 min. Absorption at 405 nm was measured using a microplate reader (Infinite M Plex, Tecan). ALP activity in mU was corrected for number of cells of each well. To assess mineralization, osteogenic differentiation was terminated after 21 days, and cells were stained with Alizarin Red S solution (2 g Alizarin Red S (02100375, MP Biomedicals, Thermo Fisher Scientific) in 60 mL water, pH 4.2) for 5 min. To determine the gene expression of osteogenic markers, RNA was harvested after four or seven days of differentiation using Trizol (15596026, Invitrogen Life Technologies) according to the manufacturer’s instructions, followed by cDNA synthesis and reverse transcriptase quantitative PCR, described in detail in the next section.

### 4.7. Reverse Transcriptase Quantitative PCR (RT-qPCR)

RNA was isolated from OS cell lines KPD, MHM, MG63, ZK58, SAOS2, U2OS, and 143B using Trizol according to the manufacturer’s instructions. cDNA synthesis was performed using iScript cDNA Synthesis Kit (1708890, Bio-rad) according to the manufacturer’s instructions. For the RT-qPCR, iQ SYBR Green Supermix (1708880, Bio-rad) and a Thermal Cycler (Bio-rad) were used with primers for osteogenic markers (*ALPL, COL1A1, SPARC, RUNX2, SPP1*), *NAMPT*, *NAPRT,* or housekeeping genes that were found to show stable expression in mesenchymal tumors (*CAPNS1, SRPR*) (Table 1) [48]. Gene expression levels’ relation to housekeeping genes were determined with the following formula: 2^^ (Ct value housekeeping genes—Ct value gene of interest)^.

### 4.8. Expression and Methylation Analysis

Methylation and gene expression data generated by the Therapeutically Applicable Research to Generate Effective Treatments (TARGET) (https://ocg.cancer.gov/programs/target (accessed on 22 February 2021)) initiative, phs000468, which include 86 osteosarcoma samples, were used in this study. Methylation data was obtained from osteosarcomas using Infinium 450 K Arrays. Probes within the complete genes, or 2000 bases around the transcription start site, of NAMPT and NAPRT1 were selected. For gene expression analysis, log2 (TPM) values of RNAseq data were used. All data used for this analysis are available on https://portal.gdc.cancer.gov/projects (accessed on 22 February 2021). For OS cell line methylation and gene expression analysis, methylation β-values and VSN-normalized gene expression data were obtained from a previously published dataset (GEO accession number GSE36002) [21]. Correlation analysis between methylation levels and gene expression was performed using R.

### 4.9. Statistical Analysis

For statistical comparisons between two groups, a Student *t*-test was performed. For multiple comparisons between groups, a Kruskal–Wallis test was used. All statistical tests were performed on the GraphPad Prism 8.

## Figures and Tables

**Table 1:** List of primer sequences used in this study.

Gene	Forward Primer	Reverse Primer
*ALPL*	TCACTCTCCGAGATGGTGGT	GCCTGCTTGGCTTTTCCTTC
*COL1A1*	AAGACGAAGACATCCCACCAAT	GTCACAGATCACGTCATCGCA
*SPARC*	CTGGACTACATCGGGCCTTG	CAGGACGTTCTTGAGCCAGT
*RUNX2*	CCCTGAACTCTGCACCAAGT	GGCTCAGGTAGGAGGGGTAA
*SPP1*	TTCGCAGACCTGACATCCAG	ACGGCTGTCCCAATCAGAAG
*NAMPT*	GGAGCATCTGCTCACTTGGT	TCATGGTCTTTCCCCCAAGC
*NAPRT*	GCTGGAGTCAGTCCTCATCG	TATAGACGCCACCCAGGGAA
*CAPNS1*	ATGGTTTTGGCATTGACACATG	GCTTGCCTGTGGTGTCGC
*SRPR*	CATTGCTTTTGCACGTAACCAA	ATTGTCTTGCATGCGGCC

## Data Availability

Data is contained within the article or Supplementary Materials.

## Supplementary Materials

The following are available online at https://www.mdpi.com/article/10.3390/ijms22126273/s1, Figure S1. (**A**) Dose response curve of FK866 in OS cell lines measured by cell viability, with IC_50_ values. (**B**) MCTS of ZK58 cells treated with FK866 with or without osteogenic medium for seven days show downregulation of osteogenic markers SPP1, RUNX2, and SPARC. Bars represent one experiment performed in triplicate ± standard deviation. (**C**) NAMPT and NAPRT1 expression in tumor tissue of osteosarcoma patients did not correlate with r^2^ = 0.007.
