# Supplementary material for: Targeting the NAD Salvage Synthesis Pathway as a Novel Therapeutic Strategy for Osteosarcomas with Low NAPRT Expression
Source: Int J Mol Sci. 2021 Jun 10;22(12):6273. doi: 10.3390/ijms22126273 (PMC8230647; doi:10.3390/ijms22126273)
Supplement: Supplementary file 1 [file ijms-22-06273-s001.zip › ijms-1228970-supplementary.pdf]

A

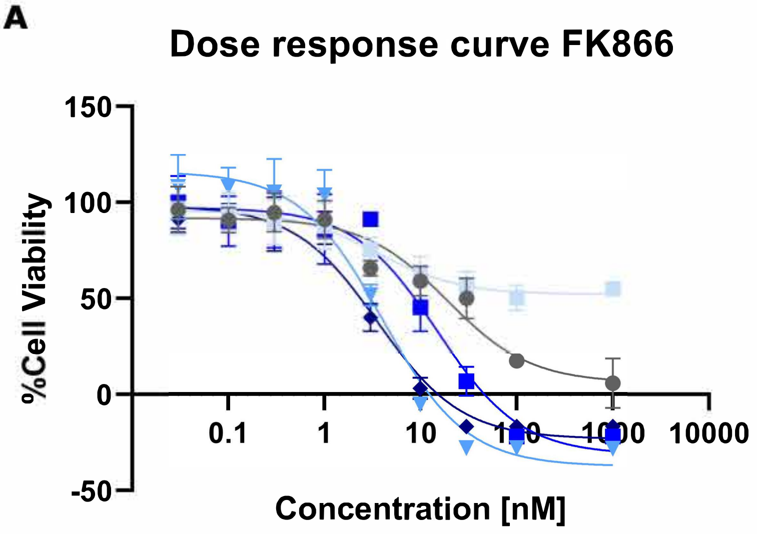

Cell line	IC50 (nM)
ZK58	19.9
MHM	N.D.
SAOS2	3.0
MG63	10.4
143BHOS	2.2

B

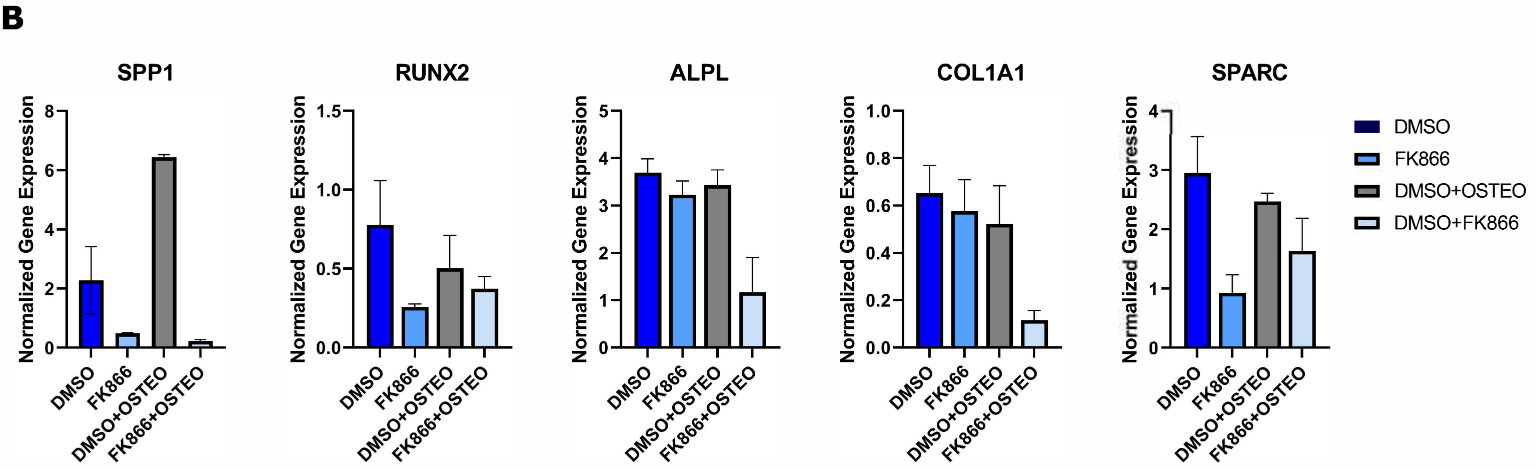

C

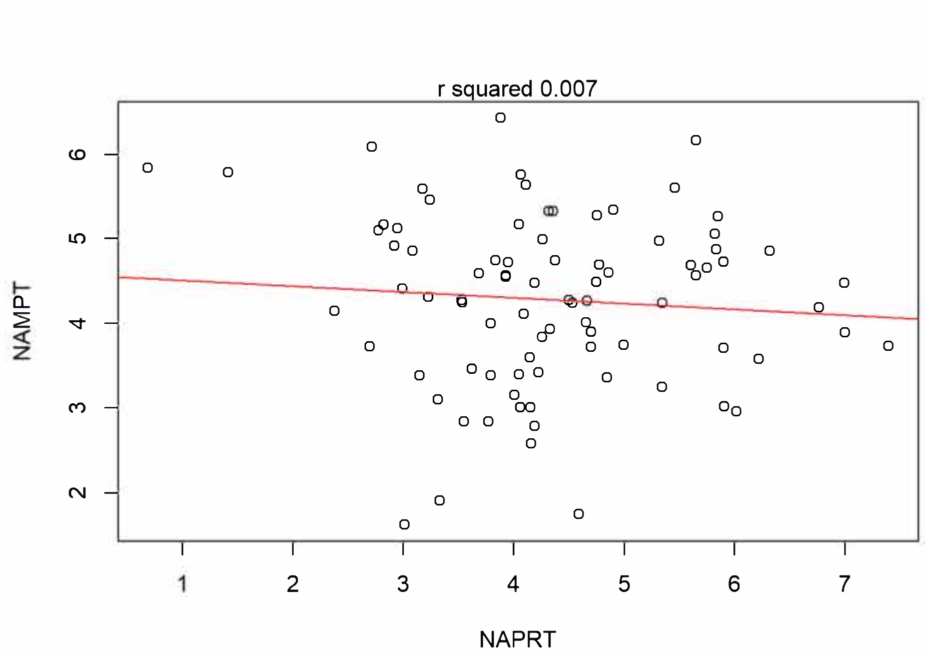

Figure S1. (**A**) Dose response curve of FK866 in OS cell lines measured by cell viability, with IC50 values. (**B**) MCTS of ZK58 cells treated with FK866 with or without osteogenic medium for seven days show downregulation of osteogenic markers SPP1, RUNX2, and SPARC. Bars represent one experiment performed in triplicate ± standard deviation. (**C**) NAMPT and NAPRT1 expression in tumor tissue of osteosarcoma patients did not correlate with r2 = 0.007.
